# Corneal Confocal Microscopy Identifies People with Type 1 Diabetes with More Rapid Corneal Nerve Fibre Loss and Progression of Neuropathy

**DOI:** 10.3390/jcm11082249

**Published:** 2022-04-18

**Authors:** Uazman Alam, Georgios Ponirakis, Omar Asghar, Ioannis N. Petropoulos, Shazli Azmi, Maria Jeziorska, Andrew Marshall, Andrew J. M. Boulton, Nathan Efron, Rayaz A. Malik

**Affiliations:** 1 Division of Diabetes, Endocrinology and Gastroenterology, Institute of Human Development, University of Manchester, Manchester M13 9PL, UK; omar.asghar@mft.nhs.uk (O.A.); shazli.azmi@manchester.ac.uk (S.A.); andrew.marshall@manchester.ac.uk (A.M.); aboulton@med.miami.edu (A.J.M.B.); 2 Department of Cardiovascular & Metabolic Medicine, Institute of Life Course and Medical Sciences, University of Liverpool, Liverpool L69 3BX, UK; 3 Department of Medicine, Weill Cornell Medicine-Qatar, Doha 24144, Qatar; gep2011@qatar-med.cornell.edu (G.P.); inp2002@qatar-med.cornell.edu (I.N.P.); ram2045@qatar-med.cornell.edu (R.A.M.); 4 Institute of Cardiovascular Science, University of Manchester, Manchester M13 9PL, UK; maria.jeziorska@manchester.ac.uk; 5 Department of Clinical Neurophysiology, Central Manchester NHS Foundation Trust, Manchester M13 9WL, UK; 6 Institute of Health and Biomedical Innovation, Queensland University of Technology, Brisbane City, QLD 4000, Australia; n.efron@qut.edu.au

**Keywords:** diabetic peripheral neuropathy (DPN), corneal confocal microscopy (CCM), Intra-epidermal nerve fiber density (IENFD), nerve conduction studies (NCS)

## Abstract

There is a need to accurately identify patients with diabetes at higher risk of developing and progressing diabetic peripheral neuropathy (DPN). Fifty subjects with Type 1 Diabetes Mellitus (T1DM) and sixteen age matched healthy controls underwent detailed neuropathy assessments including symptoms, signs, quantitative sensory testing (QST), nerve conduction studies (NCS), intra epidermal nerve fiber density (IENFD) and corneal confocal microscopy (CCM) at baseline and after 2 years of follow-up. Overall, people with type 1 diabetes mellitus showed no significant change in HbA1c, blood pressure, lipids or neuropathic symptoms, signs, QST, neurophysiology, IENFD and CCM over 2 years. However, a sub-group (*n* = 11, 22%) referred to as progressors, demonstrated rapid corneal nerve fiber loss (RCNFL) with a reduction in corneal nerve fiber density (CNFD) (*p* = 0.0006), branch density (CNBD) (*p* = 0.0002), fiber length (CNFL) (*p* = 0.0002) and sural (*p* = 0.04) and peroneal (*p* = 0.05) nerve conduction velocities, which was not related to a change in HbA1c or cardiovascular risk factors. The majority of people with T1DM and good risk factor control do not show worsening of neuropathy over 2 years. However, CCM identifies a sub-group of people with T1DM who show a more rapid decline in corneal nerve fibers and nerve conduction velocity.

## 1. Introduction

Diabetic peripheral neuropathy (DPN) affects 50% of patients with diabetes [1] and is an established risk factor for lower limb amputation [2]. It is also one of only three independent risk factors for increased mortality in patients with Type 2 diabetes mellitus (T2DM) undergoing more intensive glycemic control [3]. A number of well-established risk factors for DPN have been documented including smoking, weight, longer duration of diabetes, poorer glycemic control, hypertension, dyslipidemia and diabetic retinopathy [4,5].

Currently a major challenge in clinical trials of new treatments for DPN is in the selection of adequately sensitive endpoints [6,7]. Neuropathic symptoms and deficits, quantitative sensory testing and nerve conduction studies (NCS) [8,9] are currently accepted endpoints for clinical trials in DPN. Furthermore, a trial duration of 12 months has been recommended [9], based primarily on historical longitudinal studies demonstrating progressive deterioration in nerve conduction velocity of ~0.5 m/s/year in both T1DM [10] and T2DM [11]. However, an improvement in glycemic control as reported in the Diabetes Control and Complications Trial (DCCT) and the Epidemiology of Diabetes Interventions and Complications (EDIC) study [12,13] and medications such as ACE inhibitors and lipid lowering agents may reduce the development and progression of DPN [14,15]. In an analysis over one year in a placebo cohort demonstrated that an improvement in HbA1c and triglycerides significantly improved the summed sural and median nerve sensory conduction velocity by 2.9–5.1 m/s [16].

There is an early reduction in intra-epidermal nerve fiber density (IENFD) [17] and the axon reflex-mediated vasodilator response [18] in patients with diabetes, demonstrating subclinical pathology. A number of studies have shown dynamic changes in small nerve fibers. Smith et al. [19] demonstrated that IENFD improved with diet and exercise in patients with pre-diabetes. In T1DM, Azmi and colleagues [20,21] have shown that simultaneous pancreas and kidney transplantation is associated with early and maintained small nerve fiber regeneration in the cornea (6–12 months), followed by an improvement in neuropathic symptoms (24 months) and peroneal nerve conduction velocity (36 months). We have shown corneal nerve fiber density (CNFD) and length (CNFL) remain stable with an improvement in branch density (CNBD) over 4 years in a cohort of T1DM patients with moderate glycemic control and good blood pressure (BP) and lipid control [22]. Recently, in 37 subjects with T1D at moderate stable glycemic control followed over 6 years, there was no change in either corneal sensitivity or corneal nerve morphology [23]. However, in a large multinational consortium study (*n* = 268 of 998 people free from DPN), CNFL showed good predictive validity at ~6 years for identifying patients at higher risk of developing DPN [24]. Lewis et al. [25] have also demonstrated that an abnormally rapid loss (≥6%/yr.) of CNFL (RCNFL) occurs in 17% of people with type 1 and 2 diabetes, and these patients are at higher risk for the development and progression of DPN.

In this study, we have undertaken detailed phenotyping of large and small nerve fibers to assess progression of DPN over 2 years in a cohort of people with T1DM. We hypothesized that a subset of participants with type 1 diabetes will show rapid corneal nerve fiber loss and progression of neuropathy.

## 2. Materials and Methods

### 2.1. Selection of Patients

Patients with T1DM (*n* = 50) and age and sex matched non-diabetic healthy control participants (C) (*n* = 16) were assessed at baseline and at two years follow-up. Participants with other causes of peripheral neuropathy excluded by a medical history and laboratory panel, e.g., vitamin B12, folate, autoimmune antibodies, TFTs, renal profile, etc., and those with a history of ocular trauma or previous ocular surgery were excluded from the study. This study was observational with no active intervention. The study was approved by the North Manchester Research Ethics committee (Ref: 09/H1006/38; Date: July 2009), and written informed consent was obtained according to the Declaration of Helsinki.

### 2.2. Anthropometric and Biochemistry Measures

Body mass index (BMI) was measured (mass (kg)/(height(m))^2^). Weight was measured with a digital scale (Seca, Hamburg, Germany) to the nearest 0.1 kg and height to the nearest 0.1 cm. Blood pressure (BP) measurements were obtained using an appropriate cuff size with the use of an automated device (Dinamap pro 100v2, GE Medical Systems, Freiburg, Germany). Two measurements of systolic and diastolic BP were made five minutes apart with the lowest reading subsequently recorded.

Routine hematological and biochemical laboratory measurements which included HbA1c, estimated Glomerular Filtration Rate (eGFR) and lipid profile (total cholesterol (T-CHL), high density lipoprotein cholesterol (HDL-C), low density lipoprotein cholesterol (LDL-C) and triglycerides (TRIG)) were collected under fasting conditions.

### 2.3. Neuropathy Assessments

All participants underwent a detailed evaluation of neuropathic symptoms using the neuropathy symptom profile (NSP) and the McGill VAS to assess the severity of painful neuropathy. Clinical neurologic deficits were assessed using the modified neuropathy disability score (NDS) [26]. Diabetic neuropathy was defined according to the Toronto criteria by the presence of an abnormality of nerve conduction and a symptom or symptoms or a sign or signs of neuropathy [27].

### 2.4. Quantitative Sensory Testing (QST)

QST included assessment of vibration perception threshold (VPT) using a Neurothesiometer (Horwell, Scientific Laboratory Supplies, Wilford, Nottingham, UK), cold sensation threshold (CST) and warm sensation threshold (WST) [28] using the method of limits with the MEDOC TSA II (Medoc, Ramat Yishai, Israel) on the dorsum of the left foot (S1 dermatome).

### 2.5. Nerve Conduction Studies (NCS)

NCS were undertaken using a Dantec “Keypoint” system (Dantec Dynamics, Bristol, UK) equipped with a DISA temperature regulator to keep limb temperature constantly between 32 °C and 35 °C. Peroneal motor and sural sensory nerves were assessed in the right lower limb by a consultant neurophysiologist. The motor study was performed using silver–silver chloride surface electrodes at standardized sites defined by anatomical landmarks, and recordings for the sural nerve were taken using antidromic stimulation over a distance of 100 mm.

### 2.6. Corneal Sensitivity

Corneal sensitivity was quantified using a non-contact corneal aesthesiometer (NCCA) (Glasgow, Caledonian University, Glasgow, UK). Our NCCA methodology has been described in previous published literature [29].

### 2.7. Corneal Confocal Microscopy (CCM)

Participants underwent examination with the Heidelberg retina tomography III in vivo corneal confocal microscope employing our established methodology for image acquisition [29]. A total of 6 CCM images per participant (3 per eye) from the sub-basal nerve plexus in the central cornea (apex) were captured in a masked fashion with a lateral resolution of ~2 mm/pixel and final image size of 400 × 400 pixels. Three corneal nerve parameters were quantified: (1) corneal nerve fiber density (CNFD) (number of main nerve fibers (no./mm^2^)); (2) branch density (CNBD) (number of nerve branches (no./mm^2^)); (3) CNFL (length of nerve fibers (mm/mm^2^)). Automated corneal nerve fiber quantification (ACCMetrics software, University of Manchester, Manchester, UK) was undertaken and consists of two steps: (1) CCM image enhancement and nerve fiber detection and (2) quantification of CNFD, CNBD and CNFL [30,31]. ACCMetrics is available to all potential collaborators solely for research purposes (non-for-profit/non-commercial) and is protected by the University of Manchester in the form of a license agreement, which can be requested online (https://sites.manchester.ac.uk/ccm-image-analysis/ (accessed on 11 April 2022)).

### 2.8. Skin Biopsy and Immunohistochemistry

Intra-epidermal nerve fiber density (IENFD) was assessed in a sub-cohort of participants (T1DM *n* = 28, controls *n* = 4) who agreed to undergo a 3 mm punch skin biopsy from the dorsum of the foot, 2 cm proximal to the second metatarsal head, after local anesthesia (1% lidocaine). Our standardized immunohistochemistry protocol has been detailed previously [32]. IENFD was quantified in accord with established criteria and expressed as number per millimeter [33].

### 2.9. Rapid Corneal Nerve Fiber Loss (RCNFL)

Previously, QST abnormal ranges have been defined as values outside the 95% confidence interval of healthy subjects [34]. The “normal range” of CNFL change over 2 years is defined as values falling within 2 standard deviations of the mean in controls. Therefore, patients who demonstrated a >2SD reduction in CNFL over 2 years were considered to have rapid nerve fiber loss (RCNFL).

### 2.10. Statistical Analysis

Statistical analyses were undertaken on Statsdirect (Statsdirect, Birkenhead, Cheshire, UK). The data are expressed as mean ± standard deviation (SD). Paired *t*-test and Mann–Whitney-U test were used to assess differences between baseline and follow-up for parametric and non-parametric data, respectively. Unpaired *t*-test and Mann–Whitney-U test were used to assess differences between the control and the T1DM group for parametric and non-parametric data, respectively. Chi squared analyses were used to assess frequencies of gender and ethnicity. A significant *p* value was considered to be ≤0.05, corrected for multiple comparison tests.

### 2.11. Sample Size

The sample size analysis was based on putative small nerve fiber decline in the cohort with diabetes. An assumption of paired groups was used to calculate the sample size considering a change in CNFD over 2 years. Recruiting a minimum of 14 participants (for the diabetic group) provided 80% power to detect a clinically meaningful change in CNFD of 7.5 nerves/mm^2^ with a standard deviation of 9 nerves/mm^2^ and an assumption of a type 1 error (α-level) of 0.05. Lewis et al. [25] previously detailed 30% RCNFL in those without overt diabetic neuropathy. Therefore, a minimum of 47 participants with diabetes were required.

## 3. Results

### 3.1. Clinical and Metabolic Data

#### 3.1.1. Baseline

The demographic and metabolic characteristics are summarized in Table 1 and Table 2. Both the control (*n* = 16) and T1DM (*n* = 50) group had a comparable age, gender distribution, BMI, TRIG, HDL-C, systolic and diastolic BP, and eGFR. Compared to the control group, the T1DM group had a significantly higher mean HbA1c (*p* < 0.0001) and a significantly lower mean T-CHL (*p* = 0.003).

#### 3.1.2. Follow-Up

There was no significant change in clinical and metabolic variables between baseline and year-2 follow-up in controls and people with T1DM, apart from a significant decrease in HbA1c and systolic BP (*p* = 0.04) in controls and diastolic BP (*p* = 0.004) and eGFR (*p* = 0.02) in T1DM.

### 3.2. Diabetic Neuropathy Assessments 

#### 3.2.1. Baseline

NDS (*p* = 0.0006), McGill visual analogue score (VAS) (*p* = 0.008) and NSP (*p* < 0.0001) were significantly higher in patients with T1DM compared to controls. CNFD, CNBD and CNFL and CST were significantly lower (*p* = <0.0001–0.0007), and vibration perception (*p* < 0.0001) and corneal sensation (NCCA, *p* = 0.02) thresholds were higher in T1DM compared to controls. WST were comparable between the two groups. IENFD was lower in T1DM but did not reach significance. Peroneal motor nerve amplitude (PMNAmp), peroneal motor nerve conduction velocity (PMNCV), sural sensory nerve amplitude (SSNAmp) and sural nerve conduction velocity (SNCV) (*p* = <0.0001–0.0003) were lower in patients with T1DM compared to healthy volunteer control participants.

#### 3.2.2. Follow-Up

There were no changes in VAS, NSP, NDS, QST (apart from CST), IENFD, NCCA or CCM parameters in either group over the 2-year follow-up.

#### 3.2.3. Rapid Corneal Nerve Fiber Loss (RCNFL)

**Figure 1:**
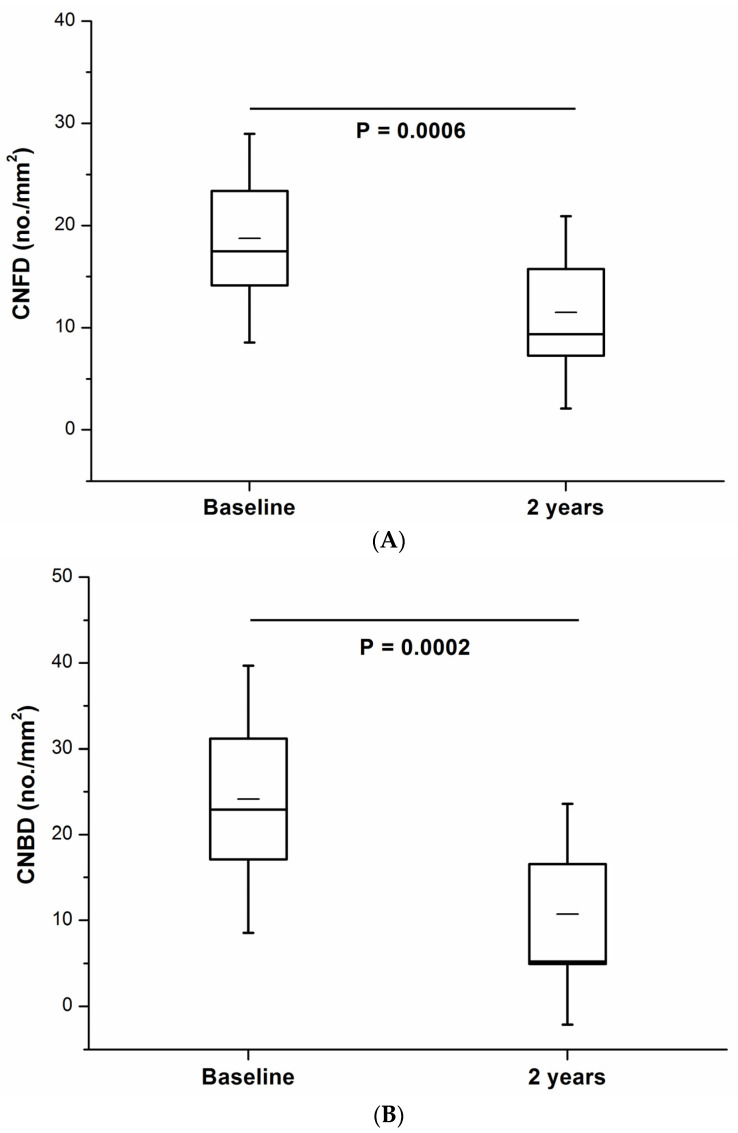

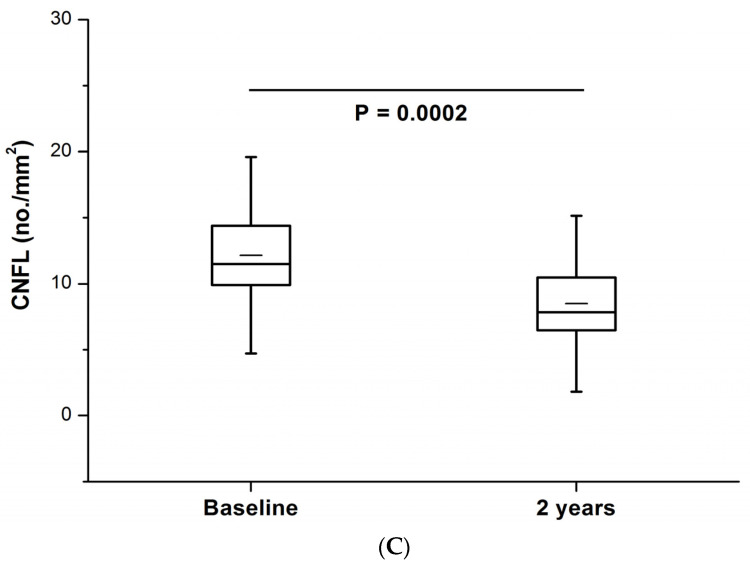
(**A**) CNFD, (**B**) CNBD and (**C**) CNFL at baseline and 2-year follow-up in the rapid corneal nerve fiber loss group (*n* = 11).

**Figure 2:**
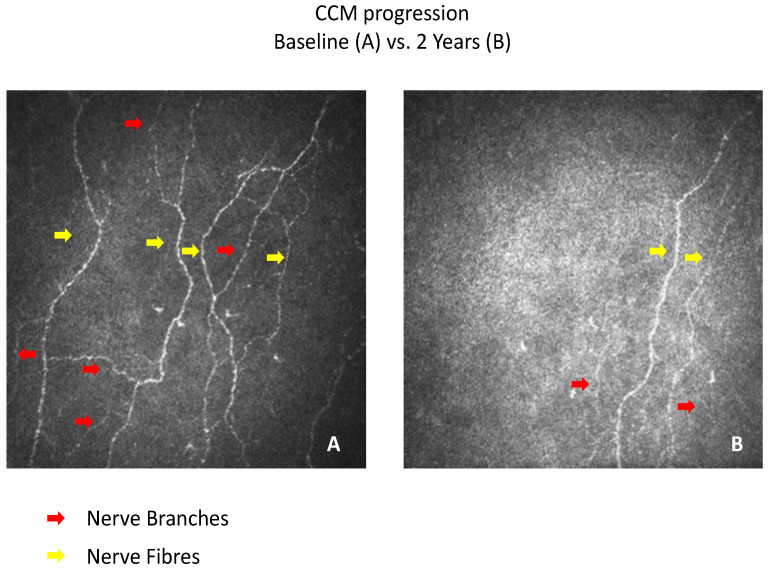
(**A**,**B**) CCM images of a subject with RCNFL at baseline and 2 year follow-up.

RCNFL was defined by a CNFL decline of >14.4%, derived from a 2 standard deviation intra-individual change over 2 years in the control group. RCNFL was identified in 22% of patients with T1DM (*n* = 11). In this sub-group, all measures of CCM were significantly lower at follow-up: CNFD (11.5 ± 9.4 vs. 18.8 ± 10.2, *p* = 0.0006), CNBD (10.7 ± 12.8 vs. 24.1 ± 15.6, *p* = 0.0002) and CNFL (8.5 ± 4.5 vs. 12.1 ± 5.0, *p* = 0.0002) (Table 3, Figure 1A–C and Figure 2A,B).

There were no significant differences between T1DM patients without (39/50) and with RCNFL (11/50) at baseline in age (47.9 ± 15.8 vs. 51.9 ± 12.4 years), duration of diabetes (28.0 ± 17.0 vs. 35.4 ± 13.5 years), HbA1c (8.3 ± 1.4 vs. 8.0 ± 0.7%), BMI (26.7 ± 4.6 vs. 27.7 ± 3.8 kg/m^2^), cholesterol-CHL (4.3 ± 1.0 vs. 4.5 ± 0.7) or systolic BP (133 ± 18 vs. 139 ± 27 mmHg).

T1DM patients with RCNFL also showed no significant change in HbA1c (7.9 ± 0.6 vs. 7.7 ± 1.0%), BMI (27.9 ± 3.3 vs. 28.8 ± 4.3 kg/m^2^), total cholesterol (4.6 ± 1.2 vs. 4.6 ± 1.3 mmol/L), systolic BP (141 ± 20 vs. 132 ± 24 mmHg) and signs or symptoms of DPN (VAS, NSP, NDS). There were no significant changes in sural (9.3 ± 8.7 vs. 6.3 ± 5.8 µV) and peroneal (2.8 ± 2.1 vs. 3.0 ± 1.5 mV) nerve amplitudes, VPT (17.5 ± 13.1 vs. 19.7 ± 14.2 volts), WST (39.2 ± 4.2 vs. 40.1 ± 4.3 °C) and IENFD (*n* = 5): 7.6 ± 4.8 vs. 6.7 ± 4.5 no/mm^2^). Although there was no statistically significant difference in CST (25.5 ± 4.0 vs. 23.3 ± 4.4 °C), the threshold value was lower (−2.2 °C) and similar to the overall cohort (−2.3 °C) (Table 2). However, there was a significant reduction in sural (42.7 ± 6.1 vs. 38.1 ± 7.0 m/s, *p* = 0.04) and peroneal (42.4 ± 3.3 vs. 41.1 ± 4.2, *p* = 0.05) NCV over 2 years.

## 4. Discussion

Our study showed no progression of diabetic neuropathy in a cohort of patients with T1DM with stable moderate glycemic control and good blood pressure and lipid control over 2 years. However, we have identified that 22% of T1DM patients show a rapid loss of corneal nerve length over 2 years which was accompanied by a reduction in sural and peroneal NCV that could not be attributed to a change in clinical, metabolic or vascular risk factors. Sural nerve conduction velocity predicts incident DPN over 4 years [35] and a number of clinical trials have utilized sural NCV as a primary endpoint [36]. In addition, peroneal nerve conduction velocity predicts both foot ulceration and death in diabetes [37].

Our study shows minimal progression of DPN [10,11,22] which is lower than previous studies demonstrating a reduction in nerve conduction velocity of ~0.5 m/s/year in T1DM [10]. Indeed, we have previously reported that CNFD or CNFL does not change, and CNBD increases over 4 years in a cohort of T1DM patients with moderate glycemic control and good blood pressure and lipid control [22]. More recently, Lewis et al. [25] showed that RCFNL was more common in people with baseline DPN, and indeed, our cohort of participants with T1DM had borderline NCS values for DPN. However, progressive corneal nerve fiber loss was not associated with baseline or change in HbA1c or BMI [25]. Lewis et al. [25] showed that rapid loss of corneal nerve fiber length appeared to be the most sensitive measure for identifying progressors [38,39]. The present study shows that a decline in CNFD, CNBD and CNFL could identify progressors. Several studies of CCM have demonstrated that a single measure of CNFL (<15 mm/mm^2^) can predict incident DPN, and those with CNFL (<11 mm/mm^2^) are likely to have prevalent DPN [38,39].

Thus, identifying patients with RCNFL may have utility in clinical trials to identify and enroll subjects with progressive rather than stable disease to enable participant enrichment for trials of new disease modifying therapies for DPN. Recent studies indicate that small nerve fiber damage, in particular a reduction in CNFL, precedes an abnormality in NCS [27,40]. Gibbons et al. reported that both NCS and QST showed no progression in patients with T2DM over 3 years, whereas there was a significant worsening in the nerve axon reflex, a measure of small fiber neuropathy [41]. In the present study, 22% of patients with T1DM showed a more rapid decline in corneal nerve fibers and peroneal and sural nerve conduction velocity. Lewis et al. showed similar outcomes and in regression analysis established a significant association between CNFL loss and other measures of diabetic neuropathy [25]. Such a rapid decline in CNFL may have clinical relevance as we have shown that it may be temporally related to the development of foot ulceration and Charcot foot [42].

Natural history studies have demonstrated early corneal nerve fiber regeneration within twelve months of simultaneous kidney and pancreas transplantation [21,43], continuous subcutaneous insulin infusion [44], and treatment with the non-erythropoietic peptide Cibenitide [45,46], with no change in other measures of neuropathy. Another study has demonstrated a significant 29% improvement in CNFL after 12 months of treatment with omega-3 polyunsaturated fatty acids, with no change in neurophysiology or QST [47]. In a randomized placebo-controlled trial of Omega-3 fatty acid in patients with type 1 diabetes, after 180 days, there was a significant increase in CNFL, but no change in thermal thresholds, autonomic function or nerve conduction studies [48]. This suggests that we either need longer clinical trial durations in excess of 3 years when utilizing current FDA-accepted endpoints of symptoms, signs and neurophysiology or that CCM could be used as a co-primary endpoint to provide an early go/no-go signal for clinical trials of disease-modifying therapies in diabetic neuropathy.

We acknowledge that the size of the cohort in this study was small, and therefore, detailed multivariate regression/correlation analyses were not possible. A future larger cohort analysis would benefit from a correlation analysis and subsequent matrix of CCM vs. NCS measures. Our data add to the current evidence that CCM can detect incident neuropathy and rapid nerve fiber decline. Indeed, long duration of diabetes and the presence of advanced neuropathy have been suggested to contribute to the failure of clinical trials. Thus, our finding that we are able to identify a sub-group of T1D patients with more rapid progression adds to the utility of CCM. These data compliment the larger studies by Lovblom et al. [38] and Lewis et al. [25], which also showed that CCM can effectively quantify neuropathy progression to risk stratify individuals and enable selection of appropriate cohorts for future clinical trials of pathogenetic treatments. Additionally, these data and previous published work also suggest that the development of a risk calculation tool incorporating CCM parameters may augment the prediction of DPN and its sequelae. A two-year follow-up is relatively short in the natural history of DPN, and a longer duration of follow-up would provide more robust results. However, the extremely detailed phenotyping of DPN has enabled us to demonstrate that people with T1DM with stable moderate glycemic control and good blood pressure and lipid control do not show the predicted deterioration in a range of measures of small and large fiber neuropathy over 2 years. CCM parameters are a more sensitive outcome measure than conventional, FDA-approved endpoints such a NCS, though future studies with larger numbers and longer follow-up intervals are warranted to verify the findings in this study. CCM is well placed to identify patients with more rapid progression of neuropathy to enable clinical trial enrichment.

## Figures and Tables

**Table 1:** Clinical and metabolic parameters in control subjects and patients with T1DM at baseline and 2-year follow-up, with statistically significant differences between groups.

	Control BL (*n* = 16)	Control FU (*n* = 16)	*p*	T1DM BL (*n* = 50)	T1DM FU (*n* = 50)	*p*
**Age (years)**	41.4 ± 11.4			48.2 ± 15.5		
**Gender (Male) (%)**	63			54		
**Duration of Diabetes (years)** **Median(IQR)**	N/a			33.1 ± 16.7		
**HbA1c (%)**	5.6 ± 0.3	5.3 ± 0.3	0.003	8.2 ± 1.3 *	8.1 ± 1.6	NS
**HbA1c (mmol/mol)**	38.0 ± 3.3	34.8 ± 3.4		66.2 ± 14.3 *	65.2 ± 17.1	
**BMI (kg/m^2^)**	25.7 ± 4.2	24.7 ± 3.8	NS	26.9 ± 4.4	27.1 ± 4.7	NS
**T-CHL (mmol/L)**	5.1 ± 0.9	4.8 ± 0.7	NS	4.3 ± 0.9 **	4.3 ± 0.9	NS
**HDL-C (mmol/L)**	1.5 ± 0.3	1.5 ± 0.3	NS	1.7 ± 0.5	1.7 ± 0.5	NS
**Triglycerides**	1.4 ± 0.7	1.2 ± 0.5	NS	1.1 ± 0.7	1.1 ± 0.5	NS
**Systolic BP (mmHg)**	125 ± 21	120 ± 19	0.02	132 ± 18	131 ± 26	NS
**Diastolic BP (mmHg)**	75 ± 12	74 ± 11	NS	73 ± 8	66 ± 9	0.004
**eGFR (mL/min/1.73)**	86 ± 7	83 ± 7	NS	81 ± 19	75 ± 17	0.02

Post hoc analyses: Control Baseline vs. T1DM Baseline; * *p* < 0.0001, ** *p* = 0.003. BMI—Body Mass Index, BP—Blood Pressure, eGFR—estimated Glomerular Filtration Rate, BL—Baseline, FU—Follow-up, HbA1c—Glycated Haemoglobin A1c, HDL-C—High Density Lipoprotein Cholesterol, NS—Non-Significant, T-CHL—Total Cholesterol.

**Table 2:** Clinical neuropathy scores and small and large fiber tests of neuropathy in control subjects and patients with T1DM at baseline and 2-year follow-up, with statistically significant differences between groups.

	Control BL (*n* = 16)	Control FU (*n* = 16)	*p*	T1DM BL (*n* = 50)	T1DM FU (*n* = 50)	*p*
**NDS (-/10)**	0.5 ± 1.1 *	0.4 ± 1.1	NS	3.4 ± 3.5 ^¥^	3.3 ± 3.6	NS
**Median (IQR)**	0 (0–0)	0 (0–0)		2 (0–6)	2 (0–6)	
**NSP (-/38)**	0.1 ± 0.25 **	0	NS	3.8 ± 5.3 ^¥¥^	4.1 ± 6.7	NS
**Median (IQR)**	0 (0–0)	0 (0–0)		1 (0–5)	1 (0–5.5)	
**McGill VAS (-/10 cm)**	0.3 ± 1.25 ^†^	0	NS	2.3 ± 3.3 ^†^	1.5 ± 3.0	NS
**Median (IQR)**	0 (0–0)	0 (0–0)		0 (0–4)	0 (0–0)	
**NCCA (mBar)**	0.6 ± 0.3	0.6 ± 0.4	NS	1.4 ± 2.3 ^††^	1.5 ± 2.3	NS
**CNFD (no./mm^2^)**	30.1 ± 4.9	28.3 ± 5.5	NS	19.5 ± 9.1 *	18.7 ± 9.9	NS
**CNBD (no/mm^2^)**	36.6 ± 15.5	39.3 ± 18.0	NS	23.9 ± 15.0 **	22.6 ± 15.8	NS
**CNFL (mm/mm^2^)**	16.9 ± 2.8	16.6 ± 3.0	NS	12.0 ± 4.6 ***	12.1 ± 5.0	NS
**CST (°C)**	28.6 ± 2.1	26.8 ± 4.8	NS	24.8 ± 6.9 ˚	22.5 ± 8.4	0.02
**WST (°C)**	37.6 ± 3.5	38.9 ± 3.8	NS	39.6 ± 4.6	40.2 ± 5.7	NS
**IENFD (no/mm)**	7.4 ± 4.8	7.4 ± 5.0	NS	5.8 ± 4.0	5.3 ± 3.5	NS
	(*n* = 4)	(*n* = 4)		(*n* = 28)	(*n* = 28)	
**VPT (volts)**	5.3 ± 4.8	6.0 ± 5.2	NS	15.5 ± 13.6 ª	15.9 ± 12.6	NS
**SSNCV (m/s)**	49.4 ± 3.9	48.1 ± 4.7	NS	43.6 ± 6.3 *^f^*	41.3 ± 6.4	NS
**SSNAmp (µV)**	21.0 ± 10.8	18.6 ± 8.7	NS	10.1 ± 6.7 *^ff^*	8.7 ± 6.8	NS
**PMNCV (m/s)**	47.8 ± 3.6	47.3 ± 4.2	NS	40.6 ± 7.0 ^^^	40.0 ± 6.6	NS
**PMNAmp (mV)**	6.0 ± 1.6	5.8 ± 1.2	NS	3.4 ± 2.4 ^^^^	3.2 ± 2.0	NS

Control Baseline vs. T1DM Baseline: ^¥^ *p* = 0.0006, ^¥¥^ *p* = <0.0001, ^†^ *p* = 0.008. ^††^ *p* = 0.02, * *p* < 0.0001. ** *p* = 0.007, *** *p* < 0.0001, ˚ *p* = 0.002, ª *p* < 0.0001, *^f^ p* < 0.0001, *^ff^ p* = 0.0003, ^^^ *p* < 0.0001, ^^^^ *p* = 0.0003. BL—Baseline, CNFD—Corneal Nerve Fiber Density, CNBD—Corneal Nerve Branch Density, CNFL—Corneal Nerve Fiber Length, CST—Cold Sensation Threshold, FU—Follow-up, IENFD—Intra-Epidermal Nerve Fiber Density, NS—Non-Significant, PMNAmp—Peroneal Motor Nerve Amplitude, PMNCV—Peroneal Motor Nerve Conduction Velocity, SSNAmp—Sural Nerve Sensory Nerve Amplitude, SSNCV—Sural Nerve Conduction Velocity, VPT—Vibration Perception Threshold, WST—Warm Sensation Threshold.

**Table 3:** People with T1DM with rapid corneal nerve fiber loss over 2 years of follow-up with statistically significant differences.

	T1DM BL (*n* = 11)	T1DM FU (*n* = 11)	*p*
**Age (years)**	54.8 ± 9.2	N/a	-
**HbA1c (%)**	7.9 ± 0.6	7.7 ± 1.0	NS
**HbA1c (mmol/mol)**	63 ± 6	61 ± 11	NS
**BMI (kg/m^2^)**	27.9 ± 3.3	28.8 ± 4.3	NS
**T-CHL (mmol/L)**	4.6 ± 1.2	4.6 ± 1.3	NS
**Systolic BP (mmHg)**	141 ± 20	132 ± 24	NS
**CNFD (no/mm^2^)**	18.8 ± 10.2	11.5 ± 9.4	0.0006
**CNBD (no/mm^2^)**	24.1 ± 15.6	10.7 ± 12.8	0.0002
**CNFL (mm/mm^2^)**	12.1 ± 5.0	8.5 ± 4.5	0.0002
**SSNCV (m/s)**	42.7 ± 6.1	38.1 ± 7.0	0.04
**SSNAmp (µV)**	9.3 ± 8.7	6.3 ± 5.8	NS
**PMNCV (m/s)**	42.4 ± 3.3	41.1 ± 4.2	0.05
**PMNAmp (mV)**	2.8 ± 2.1	3.0 ± 1.5	NS

BMI—Body Mass Index, BP—Blood Pressure, NS—Non-Significant, PMNAmp—Peroneal Motor Nerve Amplitude, PMNCV—Peroneal Motor Nerve Conduction Velocity, SSNAmp—Sural Sensory Nerve Amplitude, SSNCV—Sural Nerve Conduction Velocity, T-CHL—Total Cholesterol, VPT—Vibration Perception Threshold.

## Data Availability

Data are available from the corresponding author on reasonable request.
